# The Effect of Periodontally Accelerated Osteogenic Orthodontics on Periodontal Phenotypes in Adult Patients With Skeletal Class II Malocclusion: A Retrospective Cohort Study

**DOI:** 10.1016/j.identj.2025.104013

**Published:** 2025-11-06

**Authors:** Fengying Yin, Mengjun Li, Sen Wang, Qian Chen, Xiaoyan Chen, Xuepeng Chen, Ganggang Qi

**Affiliations:** Stomatology Hospital, School of Stomatology, Zhejiang University School of Medicine, Zhejiang Provincial Clinical Research Center for Oral Diseases, Key Laboratory of Oral Biomedical Research of Zhejiang Province, Cancer Center of Zhejiang University; Hangzhou, China

**Keywords:** Periodontally accelerated osteogenic orthodontics, Periodontal phenotypes, Skeletal Class II malocclusion, Orthodontics

## Abstract

**Aims:**

To assess the impact of periodontally accelerated osteogenic orthodontics (PAOO) on periodontal phenotypes of mandibular anterior teeth in skeletal Class II malocclusion patients.

**Methods:**

In this study, we enrolled 30 skeletal Class II malocclusion patients with 176 mandibular anterior teeth who were recommended for PAOO surgery due to insufficient labial alveolar bone in the anterior mandible. Participants were divided into two groups: the PAOO group (n = 15, receiving orthodontics with PAOO) and the control group (n = 15, receiving orthodontics alone), based on whether they had a history of PAOO or not. Data on patient characteristics, periodontal phenotypes, alveolar bone status, and dental parameters were collected from the medical record system and cone-beam CT scans at baseline (T0) and 1-year post-treatment (T1).

**Results:**

At T1, the PAOO group demonstrated significant improvements in periodontal parameters compared to the control group. The keratinized gingival width (KGW) increased by 0.76 ± 1.03mm (–0.53 ± 0.97mm in controls, *P < .*001) and gingival thickness (GT) increased by 0.38 ± 0.82mm (–0.08 ± 0.60mm in controls, *P < .*001). Gingival recession (GR) showed a minor reduction of 0.06 ± 0.71mm in the PAOO group, contrasting with an increase of 0.18 ± 0.41mm in controls (*P < .*001). The PAOO group exhibited significantly greater buccal bone height (BBH) augmentation (2.90 ± 3.77mm vs. –3.56 ± 4.48mm in controls, *P < .*001). Furthermore, buccal bone thickness (BBT) at 4mm, 6mm, and 8mm from the cemento-enamel junction and apex level showed substantial increases in the PAOO group (0.59 ± 0.90mm, 1.09 ± 0.97mm, 1.08 ± 0.97mm, and 0.61 ± 1.17mm, respectively) compared to the control group (–0.39 ± 0.51mm, –0.11 ± 0.51mm, 0.11 ± 1.01mm, and 0.05 ± 1.55mm, *P < .*01). Univariate regression analysis revealed an inverse correlation between KGW increment and preoperative KGW and GT values.

**Conclusions:**

PAOO may have the effect of improving periodontal phenotypes in skeletal Class II malocclusion patients compared to conventional orthodontics, as evidenced by improvements in KGW, GT, BBT, BBH, and GR. The treatment appears particularly beneficial for patients with thin gingival biotype or more pronounced bone deficiencies.

**Clinical Relevance:**

Traditional orthodontics presents substantial periodontal risks in the mandibular anterior region for patients with skeletal Class II malocclusion. Our study found that PAOO attenuates the detrimental periodontal consequences associated with conventional orthodontics, including keratinized tissue thinning, width reduction, and alveolar bone loss in the mandibular anterior region. Notably, the therapeutic superiority of PAOO was particularly evident in patients presenting with thin gingiva biotype or severe bone defects. These findings provide valuable evidence for developing interdisciplinary periodontal-orthodontic treatment strategies in skeletal Class II malocclusion cases.

## Introduction

The growing demand for adult orthodontic treatment has brought periodontal risk factors during therapeutic procedures into sharp focus.^1^, ^2^, ^3^ Particularly concerning are age-related oral environmental alterations and the diminished rate of alveolar bone remodeling, which predispose adult patients to periodontal complications such as gingival recession (GR), alveolar fenestration, and dehiscence during orthodontic tooth movement.^4^^,^^5^ Patients with skeletal Class II malocclusion, characterized by mandibular retrusion, maxillary protrusion, or a combination of both, represent a prevalent subgroup in orthodontic practice, with prominent lateral facial profiles frequently serving as their primary concern.^6^ In such cases, excessive proclination of the lower anterior teeth combined with the critically reduced alveolar bone thickness (BT) significantly elevates the incidence of alveolar fenestration in the mandibular anterior region.^7^ Notably, strong correlations have been observed between lower incisor proclination and the occurrence of both alveolar dehiscence and GR, which may substantially constrain the permissible range of orthodontic tooth movement.^8^ These anatomical limitations often necessitate the integration of orthodontic-orthognathic combined therapy or alveolar augmentation techniques to broaden the scope of achievable malocclusion corrections.^9^

First proposed in 2001, periodontally accelerated osteogenic orthodontics (PAOO) has been widely used in interdisciplinary periodontal-orthodontic therapy.^10^ This innovative technique combines corticotomy via punctate alveolar decortication or linear osteotomy with bone grafting, strategically designed to achieve three primary objectives: (1) acceleration of orthodontic tooth movement, (2) mitigation of periodontal risks, and (3) enhancement of post-treatment stability.^11^, ^12^, ^13^ Systematic reviews substantiate PAOO's superior efficacy over conventional orthodontics and simple corticotomy procedures, demonstrating its capacity to significantly augment alveolar BT while establishing biomechanically favorable environments for controlled dental repositioning.^14^^,^^15^

The periodontal phenotype encompasses the morphofunctional characteristics of gingival and alveolar bone tissues, representing a composite profile of periodontal hard and soft tissue architecture. As delineated in the 2017 World Workshop consensus report, the periodontal phenotype is mainly determined by the gingival phenotype and bone morphology, demonstrating dynamic adaptability to environmental factors and therapeutic interventions.^16^ Structurally, the gingival phenotype encompasses two key parameters: gingival thickness (GT) and keratinized gingival width (KGW), while the bone morphology is quantified through labial alveolar bone. Clinically significant classifications categorize gingival phenotypes into three types: thin scalloped, thick flat, and thick scalloped, which has an important impact on the health status of the periodontal tissue and the response to orthodontic treatment.^17^

Currently, research on the periodontal phenotype indicators associated with PAOO remains limited, particularly in the context of clinical studies involving patients with skeletal Class II malocclusion. Pan *et al.*^18^ found that in combined orthodontic-orthognathic treatments, the adjunctive application of PAOO did not significantly affect plaque index, probing depth (PD), bleeding index, or GR. However, it was effective in promoting the augmentation of both periodontal hard and soft tissues, facilitating the transformation from thin to thick gingiva, thereby reducing the risk of periodontal complications. In a self-controlled before-and-after study, Han *et al.*^19^ assessed changes in periodontal soft tissues following PAOO in patients with skeletal Class III malocclusion. They reported increases in KGW and GT six months post-surgery compared to pre-surgery measurements, with more pronounced tissue gains observed at sites that initially presented with thin gingiva. Another study noted no significant difference in KGW between the PAOO group and the conventional orthodontic group six months post-surgery, but confirmed that PAOO treatment enhanced alveolar BT, proving its safety and efficacy.^20^ These findings suggest variability in the outcomes related to KGW changes following PAOO surgery, with a predominant focus on alterations in alveolar BT and bone height (BH). Although PAOO primarily targets periodontal hard tissues, the effects of intraoperative procedures and postoperative responses on periodontal soft tissues are also significant and warrant attention.

Employing a retrospective cohort design, this study enrolled skeletal Class II malocclusion patients presenting with diminished alveolar BT in the lower anterior region. We evaluated the related indicators of the periodontal phenotype after orthodontics with and without PAOO and explored the related factors. This will help clinicians to better predict the PAOO outcome, ultimately informing evidence-based strategies for interdisciplinary periodontal-orthodontic management in patients with skeletal Class II malocclusion.

## Methods

This study has been reviewed and approved by the Ethics Committee of the Stomatology Hospital, Zhejiang University, School of Medicine (approval number: 2022-171 (R)), and conducted in accordance with the Helsinki Declaration of 1975, as revised in 2013.All patients have signed the informed consent before treatment.

### Patient selection

The study cohort comprised patients referred for PAOO by the Orthodontics Department between 2021 and 2022, presenting with critically thin alveolar bone volume in the mandibular anterior region. Inclusion criteria were defined as follows: (1) Diagnosis of skeletal Class II malocclusion with ANB angle > 4.7°; (2) Age ≥ 18 years old; (3) Good periodontal health; (4) No history of smoking; (5) CBCT showing dehiscence or fenestration in the lower anterior regions; (6) Cone beam computed tomography (CBCT) evidence demonstrating labial BT <1 mm at 6 mm below the cementoenamel junction (CEJ) in the mandibular anterior region; (7)Availability of complete clinical records with a minimum follow-up duration of 12 months. Exclusion criteria included: (1) Uncontrolled periodontal disease; (2) Pregnancy or lactation status; (3) Presence of prosthetic restorations or previous periodontal surgical interventions in the mandibular anterior region; (4) History of systemic conditions known to adversely affect bone metabolism or healing capacity.

### Sample size calculation

The sample size calculation was performed using G*Power 3.1 software. Based on the data from Wilcko *et al*.'s study, which reported a KGW increment of 0.78 mm in the PAOO group and -0.38 mm in the conventional orthodontics group,^21^ we determined the required sample size. With a significance level (α) set at 0.05, power (1-β) at 80%, and a 1:1 allocation ratio between groups, the minimum sample size required for this study was calculated to be 15 cases per group.

### PAOO surgery and orthodontic treatment

**Fig. 1:**
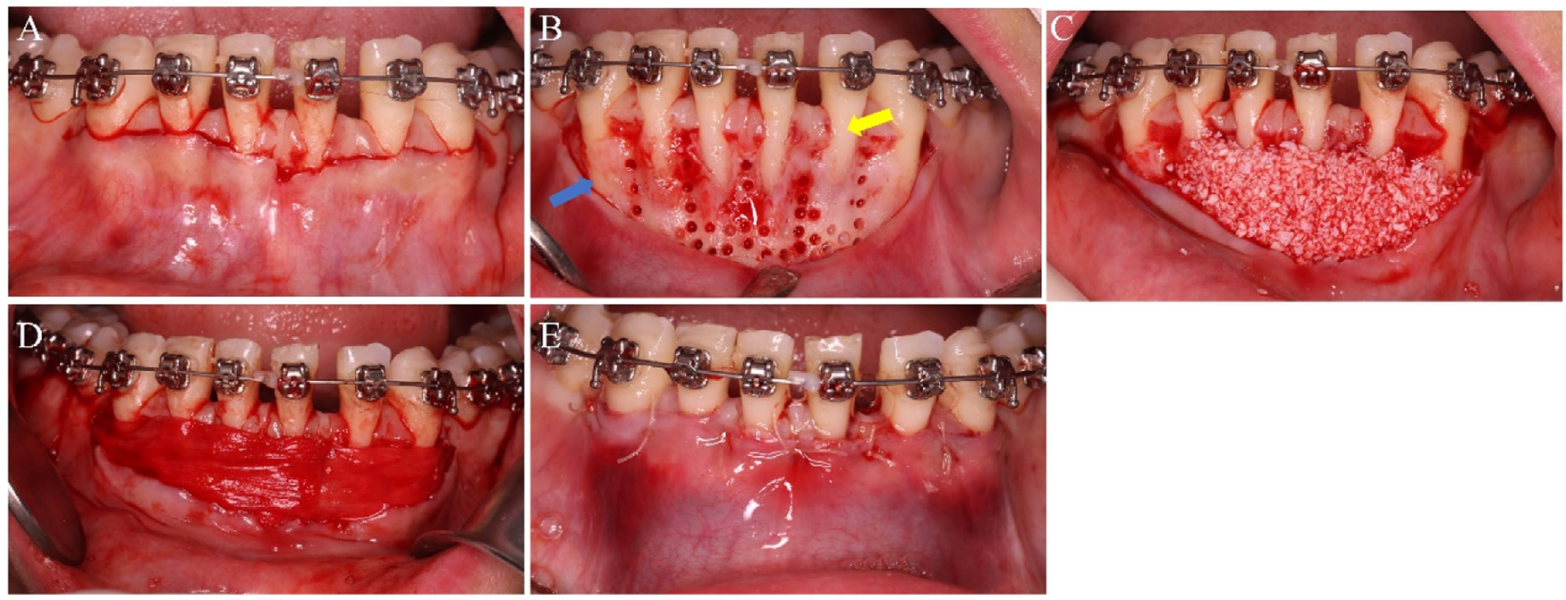
The surgical procedures. (A) Modified incision preserving the gingival papilla. (B) Perforated corticotomy cuts. The yellow arrow indicates bone fracture, and the blue arrow indicates bone fenestration. (C) Placement of bone graft material. (D) Placement of collagen membrane. (E) Horizontal mattress suture and counterpoint interrupted suture.

All enrolled patients underwent standard periodontal basic treatment, coupled with personalized oral hygiene instructions prior to orthodontic intervention. Throughout the study period, patients received periodontal support treatment at 3-month intervals. In the PAOO group, surgical procedures (Figure 1) were uniformly performed by an experienced periodontist (*Dr.* Ganggang Qi). The control group received conventional orthodontic treatment without surgical intervention. The PAOO surgical protocol was standardized as follows: A horizontal incision with papillary preservation was made extending from tooth #33 to #43. To optimize surgical access and minimize flap tension, bilateral 3-mm oblique releasing incisions were made at 45°angles distal to the canine teeth. Corticotomy was performed using piezosurgery, creating precise inter-radicular perforations through cortical plates into the medullary bone while maintaining minimal tissue trauma. Particulate bone graft material (Bio-Oss®, Geistlich, Switzerland) was placed on the labial side, ensuring a minimum thickness of 2 mm at the apical level. The graft was subsequently protected with a resorbable collagen membrane (Bio-Gide®, Geistlich, Switzerland). Part of the epithelia of the gingival papilla was removed, and the gingival flap was coronally reduced. The procedure was then completed by suturing the horizontal incisions with 5-0 absorbable sutures. Stitches were removed at 2 weeks after surgery. Fifteen patients received fixed orthodontic treatment, while fifteen others received invisible orthodontic treatment. Orthodontic forces were applied 2 weeks following periodontal surgery. ​Fixed orthodontic group:​​ Patients were treated with self-ligating bracket systems. Orthodontic adjustments were performed every 2 weeks after surgery. Superelastic nickel-titanium wires were used for initial alignment and leveling, followed by stainless steel wires for torque control. ​Invisible orthodontic group:​​ Patients were managed using the Angelalign clear aligner system. Aligners were worn as prescribed and replaced every 4 to 5 days for a 3-month period starting from the surgery date.

### CBCT examinations

Comprehensive clinical examinations and CBCT were conducted at two time points: T0 and T1. Lateral cephalogram and CBCT were retrieved from the institutional database. CBCT imaging was performed using a standardized protocol with a dedicated CBCT system (NewTom VG, Italy) under the following parameters: 110 kV, 0.3 mm scan thickness, 15 × 12 cm field of view. The effective radiation dose for each CBCT scan ranged between 35-46 microsieverts, which complies with the as low as reasonably achievable principle and radiation safety standards established by the International Commission on Radiological Protection.

### Alveolar hard and soft tissue measurements

Comprehensive clinical parameters, including PD, KGW, GR, GT, and tooth mobility (TM) were systematically recorded at both T0 and T1 time points. For radiographic analysis, CBCT datasets were processed using Dolphin Imaging software (version 11.9, Dolphin Imaging & Management Solutions, Chatsworth, USA) for three-dimensional reconstruction and spatial calibration. The reference planes were established as follows: horizontal plane: Frankfurt horizontal plane; sagittal plane: perpendicular to the horizontal plane, passing through nasion and sella turcica; coronal plane: perpendicular to both horizontal and sagittal planes, intersecting at sella turcica. The measurement plane for mandibular anterior teeth was determined by capturing the maximum mesiodistal diameter in cross-sectional view (Supplementary Figure 1). The horizontal reference line was aligned with the CEJ, while the vertical reference line connected the midpoint of the CEJ line to the root apex. Radiographic measurements, adapted from the methodology described by Ma *et al.*^11^ included: BT, labial and lingual measurements at 4 vertical levels: 4 mm, 6 mm, and 8 mm apical to CEJ, and at the root apex; BH: vertical distance from alveolar crest to CEJ; Root length: distance from CEJ to root apex. All measurements were performed on sagittal reconstructed images, as illustrated in Supplementary Figure 2, providing a comprehensive quantitative assessment of the dentoalveolar complex. To assess inter-examiner agreement and measurement reliability for CBCT images, two calibrated investigators (Mengjun Li and Fengying Yin) performed all measurements in duplicate. Prior to measurement, both investigators received standardized training from an experienced radiologist (Dr. Jun Wang, Stomatology Hospital, Zhejiang University). Excellent reliability was confirmed, with intra-class correlation coefficients exceeding 0.90 for all measurements.

### Measurement of tip and torque in mandibular anterior teeth

This study employed Dolphin Imaging software integrated with CBCT data to measure tooth tip and torque. The tooth's long axis was defined as the line connecting the crown center point (C point) and the root center point (R point). Within the 3D interface, the C point (Supplementary Fig. 3) was defined by the intersection of planes that (1) bisect the crown's labiolingual dimension in the sagittal plane, (2) bisect its mesiodistal dimension in the coronal plane, and (3) bisect its occluso-gingival dimension in the horizontal plane. The R point was defined at the position of maximum root dimension in mesiodistal and labiolingual directions, specifically located 2/5 of the root length apical to the alveolar crest. The mandibular arch was determined on the cross-sectional plane at C point level, with its outline formed by connecting the midpoint between teeth 31 and 41, tooth 43, tooth 45, and the distal point of tooth 47, depicted by a long green arc in Supplementary Figure 3. The software subsequently generated tip and torque values for target teeth automatically, with changes calculated as T1 - T0.

### Statistical analysis

Statistical analyses were performed using SPSS Statistics (version 25.0; IBM Corporation, Armonk, USA) and GraphPad Prism (version 9.5; GraphPad Software, Inc., USA). The normality of continuous variables was assessed using the Kolmogorov-Smirnov test, while homogeneity of variance was evaluated through Levene's test. Normally distributed data were presented as mean ± standard deviation (x̄ ± s), and non-normally distributed data were expressed as median with interquartile range [M (Q1, Q3)]. Parametric tests were applied for normally distributed data: paired t-tests for within-group comparisons and independent samples t-tests for between-group analyses. Non-parametric alternatives were employed for categorical variables and non-normally distributed data: Wilcoxon signed-rank test for paired comparisons and Mann-Whitney U test for independent group comparisons. A two-tailed α level of 0.05 was established as the threshold for statistical significance.

## Results

### Patient characteristics

The demographic and clinical characteristics of the study participants are presented in Supplementary Table 1. A total of 30 patients diagnosed with skeletal Class II malocclusion were enrolled in this study, consisting of 11 male (36.7%) and 19 female (63.3%) participants. Participants were allocated into two groups: PAOO group (n = 15): 5 males (33.3%) and 10 females (66.7%), average age 26.20 ± 6.19 years; Control group (n = 15): 6 males (40.0%) and 9 females (60.0%), average age 27.67 ± 6.39 years. Fifteen patients were treated with fixed appliances, and the remainder with invisible appliances. There was no significant difference in the ratio of appliances between the two groups (*P = .*273). Baseline assessments revealed no statistically significant intergroup differences in periodontal parameters, appliance selection, or cephalometric measurements (SNA, SNB, and ANB angles). The study evaluated a total of 176 teeth, distributed as: PAOO group:30 central incisors +28 lateral incisors (2 congenitally missing)+30 canines; Control group:28 central incisors (2 congenitally missing)+30 lateral incisors +30 canines.

### Periodontal outcomes

The periodontal clinical indicators for both the PAOO and control groups were comprehensively summarized in Table 1, encompassing measurements taken before treatment and at 1-year post-treatment. At baseline, no significant differences were observed between the PAOO and control groups in terms of PD, KGW, GT, GR, and TM. Throughout the study, PD remained stable in both groups. In the PAOO group, KGW and GT showed an increase at T1 (ΔKGW: 0.76 ± 1.03mm; ΔGT:0.38 ± 0.82mm), whereas these parameters decreased in the control group (ΔKGW:-0.53 ± 0.97mm; ΔGT:-0.08 ± 0.60mm, all *P < .*001). Although GR was more obvious in the control group (0.18 ± 0.41mm), there was only a slight difference compared with the PAOO group (-0.06 ± 0.71mm, *P < .*001). Additionally, TM improved in the PAOO group at T1, whereas no significant change was observed in the conventional orthodontic group. These findings demonstrate that PAOO treatment had notable improvements in KGW, GT, and TM at 1-year post-treatment compared to conventional orthodontics, suggesting that PAOO serves as an effective intervention for enhancing the periodontal health of orthodontic patients.

**Table 1:** Comparison of periodontal clinical indicators before treatment (T0) and at 1-year post-treatment (T1).

Periodontal clinical indicators		PAOO group (n = 88)	Conventional orthodontic group (n = 88)	T* or Z*	P*
PD/mm $\bar{x}\pm s$	T0	1.80 ± 0.67	1.83 ± 0.68	−0.453	.651
	T1	1.74 ± 0.62	1.80 ± 0.64	−1.031	.303
	Δ PD	−0.06 ± 0.80	−0.03 ± 0.76	−0.446	.656
	*t*†	1.148	0.569		
	*P*†	.205	.570		
GR/mm $\bar{x}\;\pm \;s$	T0	0.14 ± 0.87	0.19 ± 0.47	−0.932	.352
	T1	0.08 ± 1.07	0.38 ± 0.68	−3.825	**.000**
	Δ GR	−0.06 ± 0.71	0.18 ± 0.41	−4.783	**.000**
	*t*†	1.345	−7.283		
	*P*†	.180	**.000**		
KGW/ mm $\bar{x}\;\pm \;s$	T0	4.09 ± 1.16	3.91 ± 1.17	1.034	.302
	T1	4.85 ± 1.19	3.38 ± 1.05	8.721	**.000**
	Δ KGW	0.76 ± 1.03	−0.53 ± 0.97	8.596	**.000**
	*t*†	−6.946	5.164		
	*P*†	**.000**	**.000**		
GT/ mm $\bar{x}\;\pm \;s$	T0	1.13 ± 0.78	1.07 ± 1.05	0.439	.661
	T1	1.50 ± 0.43	0.99 ± 0.93	4.708	**.000**
	Δ GT	0.38 ± 0.82	−0.08 ± 0.60	4.164	**.000**
	*t*†	−4.287	1.199		
	*P*†	**.000**	.234		
TM M (*P*_25,_*P*_75_)	T0	0 (0,1)	0 (0,1)	−0.007	.994
	T1	0 (0,0)	0 (0,1)	−2.659	**.008**
	*Z* value†	−3.272	1.890		
	*P*†	**.001**	.059		

PAOO, Periodontally accelerated osteogenic orthodontics; PD, Probing depth; GR, Gingival recession; KGW, Keratinized gingival width; GT, Gingival thickness; TM, Tooth mobility; Δ, T1-T0.

⁎ denotes the comparison between the PAOO group and the conventional orthodontic group.

† denotes the comparison between T1 and T0.

### Radiographic outcomes

The data pertaining to BT and BH on labial/lingual sides, as well as root length measurements at T0 and T1, are systematically presented in Table 2 for both PAOO and control groups. At 1-year after surgery, the labial BT at four predefined distances from the CEJ were all significantly increased in the PAOO group. In contrast, the conventional orthodontics group demonstrated significant labial BT reduction only at the 4mm under CEJ (BBT 4mm), with no notable changes at other sites. Comparative analysis showed superior buccal bone augmentation on the buccal side in the PAOO group relative to controls. For lingual BT measurements, neither group exhibited significant changes at 4mm (LBT 4mm), 6mm (LBT 6mm) from CEJ, or root apex regions. However, both groups displayed significant BT increases at the 8mm from CEJ (LBT 8mm). The control group experienced progressive loss in BBH (3.56 ± 4.48mm), whereas the PAOO group showed significant BBH improvement from T0 to T1 (–2.90 ± 3.77mm, *P < .*001), suggesting PAOO's potential to counteract labial bone resorption associated with conventional orthodontic therapy. At the 1-year post-treatment, the loss of lingual bone height (ΔLBH) in the PAOO group was 0.50 ± 2.54mm, compared to 0.73 ± 2.61mm in the control group. The greater LBH loss observed in the control group suggests that PAOO may also confer benefits for the lingual alveolar bone. However, ​no statistically significant difference​ was found between the two groups (*P = .*553). At the end of follow-up, the root length was significantly shorter in both groups. Although the root absorption in the PAOO group (0.27 ± 0.51mm) was slightly lower than that in the conventional orthodontics group (0.35 ± 0.78mm), the difference was not statistically significant, as shown in Table 2.

**Table 2:** Changes in alveolar bone height, thickness, and root length before (T0) treatment (T0) and at 1-year after treatment (T1).

		PAOO group (n = 88)	Conventional orthodontic group (n = 88)	t*	P*
BBT 4mm	T0	0.45 ± 0.65	0.60 ± 0.49	−1.773	.078
	T1	1.04 ± 0.93	0.21 ± 0.37	7.715	**.000**
	ΔBBT 4mm	0.59 ± 0.90	−0.39 ± 0.51	8.886	**.000**
	*t*†	−6.175	7.119		
	*P*†	**.000**	**.000**		
BBT 6mm	T0	0.48 ± 0.65	0.34 ± 0.46	1.666	.097
	T1	1.57 ± 0.94	0.23 ± 0.42	12.167	**.000**
	ΔBBT 6mm	1.09 ± 0.97	−0.11 ± 0.51	10.197	**.000**
	*t*†	−10.485	1.957		
	*P*†	**.000**	.054		
BBT 8mm	T0	0.84 ± 0.80	0.84 ± 0.74	0.026	.979
	T1	1.93 ± 0.85	0.95 ± 1.08	6.704	**.000**
	ΔBBT 8mm	1.08 ± 0.97	0.11 ± 1.01	6.508	**.000**
	*t*†	−10.352	−0.980		
	*P*†	**.000**	.330		
BBT apical	T0	2.67 ± 1.40	3.27 ± 1.09	−3.187	**.002**
	T1	3.28 ± 1.18	3.32 ± 1.68	−0.187	.852
	ΔBBT apical	0.61 ± 1.17	0.05 ± 1.55	2.708	**.007**
	*t*†	−4.867	−0.286		
	*P*†	**.000**	.776		
LBT 4mm	T0	1.23 ± 1.02	1.42 ± 1.03	−1.265	.208
	T1	1.17 ± 0.94	1.40 ± 1.00	−1.609	.109
	ΔLBT 4mm	−0.06 ± 0.91	0.02 ± 0.96	−0.283	.778
	*t*†	0.575	0.153		
	*P*†	.567	.879		
LBT 6mm	T0	1.78 ± 1.12	1.88 ± 1.13	−0.631	.529
	T1	1.78 ± 1.26	1.87 ± 1.25	−0.448	.655
	ΔLBT 6mm	0.00 ± 0.72	−0.02 ± 1.02	0.167	.867
	*t*†	−0.040	0.177		
	*P*†	.968	.860		.076
LBT 8mm	T0	2.15 ± 1.27	2.38 ± 1.21	−1.235	.219
	T1	2.36 ± 1.54	2.73 ± 1.88	−1.423	.157
	ΔLBT 8mm	0.21 ± 0.94	0.34 ± 1.51	−0.732	.465
	*t*†	−2.108	−2.175		
	*P*†	**.038**	**.032**		.465
LBT apical	T0	4.23 ± 1.60	4.82 ± 1.71	−2.349	**.02**
	T1	4.50 ± 1.97	4.74 ± 2.15	−0.778	.438
	ΔLBT apical	0.27 ± 1.26	−0.08 ± 1.84	1.440	.152
	*t*†	−1.966	0.397		
	*P*†	.052	.692		
BBH	T0	5.84 ± 3.93	3.11 ± 2.71	5.362	**.000**
	T1	2.94 ± 2.03	6.67 ± 3.60	−8.462	**.000**
	Δ BBH	−2.90 ± 3.77	3.56 ± 4.48	−10.336	**.000**
	*t*†	7.214	−7.439		
	*P*†	**.000**	**.000**		
LBH	T0	3.13 ± 2.25	2.20 ± 2.10	2.815	.005
	T1	3.63 ± 2.51	2.93 ± 2.94	1.679	.095
	Δ LBH	0.50 ± 2.54	0.73 ± 2.61	−0.595	.553
	*t*†	−1.840	−2.621		
	*P*†	.069	**.01**		
RL	T0	11.53 ± 1.06	11.82 ± 1.14	−1.764	.079
	T1	11.27 ± 1.11	11.47 ± 1.31	−1.138	.257
	Δ RL	−0.27 ± 0.51	−0.35 ± 0.78	0.853	.395
	*t*†	4.921	4.222		
	*P*†	**.000**	**.000**		

BBT 4mm, 6mm, and 8mm represent the alveolar bone thickness at 4mm, 6mm, and 8mm apical to CEJ on the buccal side, and LBT 4mm, 6mm, and 8mm represent the corresponding measurements on the lingual side.

BBH (buccal bone height) and LBH (lingual bone height) respectively represent the distance from the alveolar crest to CEJ on the buccal and lingual side. RL, root length; Δ, T1-T0.

⁎ Denotes the comparison between the PAOO group and the conventional orthodontic group;

† Denotes the comparison between T1 and T0.

### Factors influencing periodontal phenotype changes following PAOO

To investigate determinants of periodontal phenotype alterations induced by PAOO, we conducted univariate regression analyses using preoperative KGW, gingival, GT, gender, age, tooth site, periodontal status, correction type, ΔTip, and ΔTorque as independent variables, with postoperative ΔKGW and ΔGT as dependent variables (Table 3). Key findings revealed: 1) ΔKGW demonstrated significant negative correlations with baseline KGW (β = –0.367, *P < .*001) and GT (β = –0.311, *P < .*05), indicating diminished surgical KGW augmentation in sites with initially wider/thicker keratinized tissues. 2) Gender disparity: Females exhibited approximately 0.5 mm greater KGW gain compared to males (*P < .*05). 3)ΔGT inversely correlated with preoperative GT (β = –0.897, *P < .*001). In addition, we also explored the factors that might influence the increment of alveolar bone. Analysis of alveolar bone dynamics (Table 4) disclosed: 1) BBT changes inversely correlated with preoperative BBT, with greater augmentation occurring at sites of initial buccal bone thinness. 2)BBH increment is positively associated with preoperative BBH, demonstrating enhanced vertical augmentation in areas with greater baseline alveolar ridge-CEJ distance. Comparative evaluation of T1-T0 torque and tip alterations revealed a significant lingual torque reduction in mandibular anterior teeth (Supplementary Table 2). The tip of the central incisors increased slightly, but the tip of the lateral incisors and canines did not change significantly. The univariate regression analysis showed that BBT was negatively correlated with ΔTip and ΔTorque at some tooth sites (Table 5). BBH was also negatively correlated with torque variation, but it was statistically significant only at the lateral incisors.

**Table 3:** Univariate linear regression analysis of factors influencing ΔKGW and ΔGT at 1-year after PAOO treatment.

Metric	Variable	β (95%CI)	SE	Standardized β	R^2^	*t*	*P*
ΔKGW	KGW	−0.367 (−0.540, −0.194)	0.087	−0.413	0.172	−4.229	**.000**
	GT	−0.311 (−0.584, −0.037)	0.138	−0.237	0.056	−2.259	**.026**
	Gender	0.498 (0.048,0.948)	0.226	0.231	0.053	2.199	**.031**
	Age	0.003 (−0.034,0.039)	0.018	0.017	0.000	0.159	.874
	Tooth Site						
	Lateral incisor	−0.174 (−0.643,0.295)	0.236	−0.079	0.006	−0.737	.463
	Canine	0.008 (−0.454,0.470)	0.233	0.004	0.000	0.035	.972
	Periodontal condition	−0.417 (−0.901,0.067)	0.243	−0.181	0.033	−1.712	.091
	Appliance type	−0.160 (−0.605,0.284)	0.224	−0.077	0.006	−0.717	.475
	ΔTip	−0.020 (−0.090,0.050)	0.035	−0.061	0.004	−0.566	.573
	ΔTorque	0.052 (−0.045,0.149)	0.049	0.115	0.013	1.070	.288
ΔGT	KGW	−0.085 (−0.065, 0.236)	0.076	0.121	0.003	1.128	.263
	GT	−0.897 (−1.013,−0.781)	0.058	−0.856	0.732	−15.338	**.000**
	Gender	0.089 (−0.280,0.457)	0.185	0.052	0.003	0.478	.634
	Age	−0.019 (−0.048,0.010)	0.014	−0.141	0.020	−1.324	.189
	Tooth Site						
	Lateral incisor	−0.089 (−0.464,0.286)	0.189	−0.051	0.003	−0.473	.638
	Canine	−0.058 (−0.427,0.311)	0.186	−0.034	0.001	−0.312	.756
	Periodontal condition	−0.023 (−0.415,0.370)	0.198	−0.012	0.000	−0.114	.910
	Appliance type	−0.099 (−0.455,0.256)	0.179	−0.060	0.004	−0.556	.580
	ΔTip	0.002 (−0.054,0.058)	0.028	0.009	0.000	0.079	.937
	ΔTorque	−0.000 (−0.077,0.078)	0.039	0.001	0.000	0.012	.991

KGW, keratinized gingival width; GT, gingival thickness; Δ, T1-T0.

**Table 4:** Correlation between changes in labial bone thickness, height, and preoperative labial bone thickness, and height at the same location.

Measurement	Central incisor		Lateral incisor		Canine	
	*R*	*P*	*R*	*P*	*R*	*P*
ΔBBT 4mm	−0.311	.094	−0.533	**.003**	−0.087	.647
ΔBBT 6mm	−0.431	**.017**	−0.238	.223	−0.481	**.007**
ΔBBT 8mm	−0.618	**.000**	−0.602	**.001**	−0.462	**.010**
ΔBBT apical	−0.672	**.000**	−0.688	**.000**	−0.491	**.006**
Δ BBH	0.817	**.000**	0.826	**.000**	0.928	**.000**

BBT 4mm, 6mm, and 8mm represent the alveolar bone thickness at 4mm, 6mm, and 8mm apical to CEJ on the buccal side**.**

BBH, buccal bone height; **Δ,** T1-T0.

**Table 5:** Correlation between labial bone changes and ΔTip, and ΔTorque at 1-year after PAOO treatment.

Variable	Measurement	Central incisor		Lateral incisor		Canine	
		R	*P*	R	*P*	R	*P*
ΔTip	ΔBBT 4mm	−0.403	**.027**	−0.410	.030*	−0.045	.814
	ΔBBT 6mm	−0.298	.110	−0.303	.117	0.249	.184
	ΔBBT 8mm	−0.039	.838	−0.480	**.031**	0.148	.436
	ΔBBT apical	0.095	.619	0.248	.204	0.055	.771
	Δ BBH	−0.088	.645	−0.172	.380	−0.099	.604
ΔTorque	ΔBBT 4mm	0.126	.507	−0.348	.069	−0.059	.757
	ΔBBT 6mm	−0.182	.335	−0.483	**.009**	−0.553	**.002**
	ΔBBT 8mm	−0.308	.098	−0.480	**.010**	−0.321	.083
	ΔBBT apical	−0.404	**.027**	−0.339	.078	−0.212	.261
	Δ BBH	−0.125	.509	−0.386	**.042**	−0.003	.988

BBT 4mm, 6mm, and 8mm represent the alveolar bone thickness at 4mm, 6mm, and 8mm apical to CEJ on the buccal side**.**

BBH, buccal bone height; **Δ**, T1-T0.

## Discussion

For patients with skeletal Class II malocclusion, severe lip inclination of lower anterior teeth, high incidence of bone fenestration, or bone cracking carry a great risk of marginal bone loss and gingival recession in orthodontic treatment. This study comprehensively compared periodontal phenotype modifications between PAOO and conventional orthodontic therapy in these patients. Key comparative outcomes demonstrated: (1) Conventional orthodontics caused gingival recession, reduced KGW, and decreased GT in the lower anterior tooth area, but PAOO intervention counteracted these adverse effects, showing improvement in soft tissue parameters. (2) Conventional treatment maintained BBT but significantly reduced BBH. PAOO achieved substantial labial bone augmentation, increasing both BBT and BBH. (3) The smaller the KGW and the thinner the GT before surgery, the larger the increment of KGW and GT caused by PAOO. (4) The thinner the alveolar bone was before operation, the greater the increment of BT obtained after PAOO. The further the alveolar crest was from CEJ before operation, the easier it was to gain bone height increment. (5) PAOO did not improve root resorption associated with traditional orthodontics. These findings are worthy of clinicians’ attention and consideration for risk avoidance by PAOO surgery. Preoperative anatomical assessment of KGW, GT, and alveolar morphology should guide surgical decision-making to optimize periodontal outcomes.

Periodontal phenotype classification is primarily determined by three key parameters: KGW, GT, and labial bone morphology, with gingival phenotypes being predominantly influenced by KGW and GT.^17^ Many researches have suggested that PAOO can improve the periodontal phenotype in patients with skeletal Class III malocclusion. However, there are few studies on patients with skeletal Class II malocclusion, particularly concerning the mechanistic understanding of postoperative periodontal phenotype alterations. This study, therefore, aims to systematically evaluate PAOO-induced periodontal phenotype modifications in skeletal Class II malocclusion patients and preliminarily analyze the possible mechanisms driving these phenotypic changes.

The significance of KGW and GT for gingival margin stability and periodontal health, particularly during orthodontic treatment, has been well-established in prior research.^17^^,^^22^^,^^23^ Consistent with this, Mascardo *et al.* demonstrated a significant association between GR and reduced KGW and GT. Specifically, each additional millimeter of KGW was associated with a 38% lower risk of GR, while each additional millimeter of GT corresponded to an 82% risk reduction.^24^ In our study, conventional orthodontic treatment resulted in reductions of both KGW and GT. In contrast, PAOO significantly enhanced these parameters, aligning with the earlier findings of Han *et al.*^19^ and Wang *et al*.^13^ Wilcko *et al.* conducted an image analysis revealing approximately 1 mm greater KGW augmentation in PAOO-treated cases compared to conventional orthodontics.^21^ In the study of Wang *et al.*, PAOO could increase KGW in patients with skeletal Class I, while Xu *et al*.'s investigation of maxillary anterior teeth in skeletal Class III cases showed no significant KGW enhancement relative to conventional treatment.^20^ These divergent outcomes may be attributed to anatomical and biomechanical variations, including differential muscular traction forces between arches, distinct tooth movement patterns, and baseline KGW discrepancies. Although the difference in GR change between the PAOO group and the control group was statistically significant, its magnitude was clinically small, indicating stable gingival margins in both groups at 1-year post-treatment evaluation. The larger standard deviation observed within the PAOO group suggests some individual variability in treatment response. This stability is also supported by a 2019 prospective cohort study involving 19 patients (692 teeth) with Angle Class I malocclusion, which reported negligible GR change (0.00 ± 0.39 mm) after an average observation period of 1.28 years, confirming PAOO's efficacy in preserving gingival margin stability.^25^

Univariate regression analysis revealed significant negative correlations between KGW augmentation and both preoperative KGW and GT, suggesting greater therapeutic benefits in patients with initially thin gingival tissues. However, the incremental effect was limited, necessitating adjunctive soft tissue augmentation procedures when there is severe gingival receding or very thin gingiva in the operative area to ensure periodontal health.^26^ Similarly, GT increment demonstrated an inverse relationship with baseline GT, potentially attributable to the expanded regenerative space created by PAOO in thin gingival phenotypes. Notably, female patients exhibited greater KGW improvement than males, possibly because the thin gingival type is more common in female patients,^27^ and estrogen can also promote gingival epithelial hyperplasia to a certain extent.^28^

A previous study noted that skeletal Class II patients showed a high prevalence of alveolar defects (41.11%) surrounding the anterior mandibular teeth.^7^ In orthodontic treatment, sufficient alveolar bone volume is crucial for preventing root exposure, GR, and even treatment relapse or failure.^29^^,^^30^ Bone augmentation is a significant advantage of PAOO and is one of the reasons why it is gradually widely used in clinical. At 1 year follow-up, PAOO demonstrated substantial increases in labial bone height (BBH) and thickness (BBT) concentrated in the apical third, consistent with Jing *et al*.'s findings.^31^ However, Wang *et al.*^13^ reported no significant difference in BT increments between PAOO and conventional orthodontic groups, potentially due to their cohort's thicker preoperative BT compared to our study and Ma *et al*.'s report.^11^ Although the pre-treatment data showed no significant differences in the thickness and height of the lingual alveolar bone, as well as the BBT at 4mm, 6mm, and 8mm under CEJ between PAOO and control groups, lower BBH and thinner apical BT did exist in the PAOO group. This aligns with the clinical rationale for PAOO, which is typically indicated for patients with a higher risk of bone defects during orthodontic tooth movement. Thus, the selection bias stems from treatment indication rather than sampling error. Due to the limitations of retrospective studies, there may also be other selection biases, such as patients' preferences for different treatment methods or expected differences in treatment effects. Future prospective studies with randomized controlled trials are needed to validate these findings.

Univariate linear regression analysis also confirmed an inverse relationship between BBT augmentation and baseline BBT, indicating enhanced bone regeneration in initially thin alveolar plates. Dentoalveolar positional analysis revealed significant lingual inclination of mandibular anterior teeth at T1, consistent with Potts *et al*.'s observations^32^ and suggesting improved occlusal relationships. Furthermore, alveolar bone augmentation showed negative correlations with torque changes, particularly in incisor regions.

There has been debate about whether PAOO increases or decreases root resorption. Root resorption is a multifactorial biological process, which is influenced by orthodontic force magnitude, patient age, anatomical variations, etc.^33^ A systematic review published in 2019 showed that existing studies did not have sufficient data to evaluate PAOO-related root resorption.^15^ While several studies have reported root resorption after PAOO,^34^^,^^35^ most studies found no significant correlation between PAOO and root resorption.^36^, ^37^, ^38^ Our findings align with the latter perspective, demonstrating no statistically significant difference in root resorption between PAOO and conventional orthodontic groups, though a trend toward reduced resorption was observed in the PAOO cohort. However, the 12-month follow-up period limits our ability to assess long-term root integrity, necessitating extended longitudinal studies.

Current understanding of factors influencing PAOO-mediated periodontal phenotype modifications remains limited. Beyond the variables examined in this study, additional determinants may include: (1) patient-specific factors such as dental anatomy and individual biological reactions; (2) surgical parameters including bone graft material selection and surgical approaches.^39^ (3) orthodontic variables related to tooth movement direction and distance. Furthermore, emerging evidence suggests sagittal and vertical skeletal relationships may significantly impact anterior alveolar dimensions,^40^^,^^41^ though these factors were not accounted for in our analysis. The retrospective cohort design and relatively small sample size represent methodological limitations that may affect the generalizability of our findings. Randomized controlled clinical studies with large samples are needed to establish robust evidence regarding PAOO's impact on periodontal phenotype and identify key predictive factors. It is important to emphasize that while the 1-year follow-up in this study provides valuable data on the early stability achieved with PAOO, longer-term follow-up remains essential to confirm sustained efficacy and identify potential late-term changes. Future studies with extended observation periods will be crucial to provide more comprehensive conclusions.

## Conclusion

In summary, our findings demonstrate significantly elevated risks of keratinized tissue thinning, width reduction, and alveolar bone resorption in the mandibular anterior region following conventional orthodontic treatment for skeletal Class II malocclusion, whereas enhanced periodontal phenotype modulation via PAOO may reduce these adverse effects. Importantly, the therapeutic superiority of PAOO was particularly pronounced in patients presenting with thin gingiva or severe bone defects. These findings collectively establish a foundation for PAOO's tissue regenerative capacity while underscoring its clinical value in high-risk anatomical profiles. Future investigations should prioritize multicenter longitudinal studies and large samples to develop predictive models for individualized risk stratification of orthodontic-induced tissue loss, thereby optimizing patient-specific treatment protocols.

## Ethics approval statement

The study protocol has been reviewed and approved by the Ethics Committee of the Stomatology Hospital, Zhejiang University, School of Medicine (No. 2022-171R).

## Patient consent statement

Informed consent was obtained from all individual participants included in the study.

## Declaration of generative AI and AI-assisted technologies in the writing process

During the preparation of this work the author(s) used [Deepseek] in order to [improve language and readability]. After using this tool, the author(s) reviewed and edited the content as needed and take(s) full responsibility for the content of the publication.

## Author contributions

All authors contributed to the study's conception and design. Material preparation, data collection, and analysis were performed by FYY, MJL, SW, QC, XYC, and XPC. GGQ administrated and supervised the project. The first draft of the manuscript was written by FYY and all authors commented on previous versions of the manuscript. All authors read and approved the final manuscript.

## Conflict of interest

The authors have no relevant financial or non-financial interests to disclose.

## Appendix Supplementary materials

Image, application 1

## Footnotes

Supplementary material associated with this article can be found in the online version at doi:10.1016/j.identj.2025.104013.
