# Supplementary material for: The Effect of Periodontally Accelerated Osteogenic Orthodontics on Periodontal Phenotypes in Adult Patients With Skeletal Class II Malocclusion: A Retrospective Cohort Study
Source: Int Dent J. 2025 Nov 6;76(1):104013. doi: 10.1016/j.identj.2025.104013 (PMC12637380; doi:10.1016/j.identj.2025.104013)
Supplement: Supplementary file 1 [file mmc1.docx]

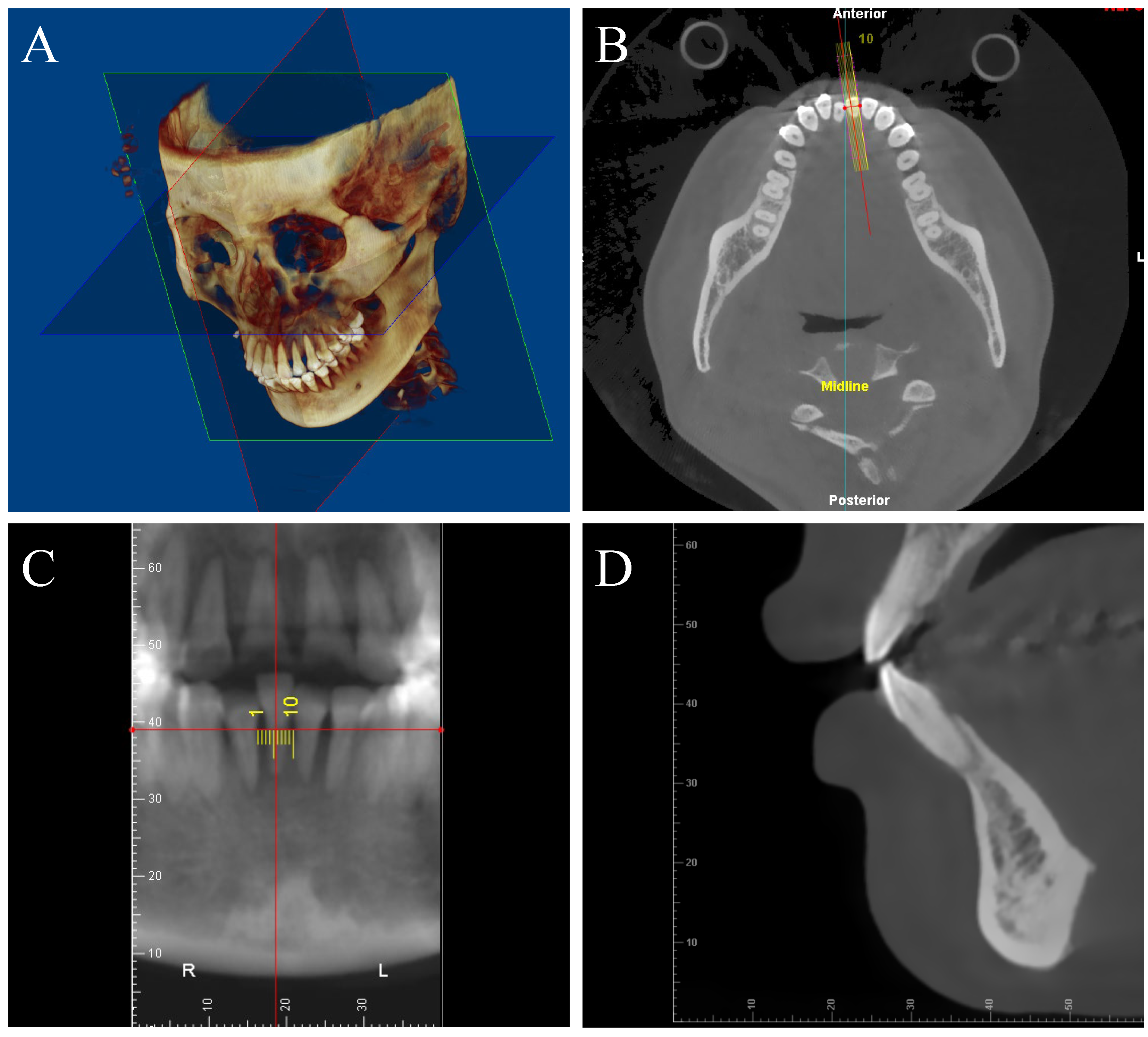


**Supplementary Figure 1**. (A) Reference plane after CBCT 3D reconstruction and calibration.

(B) In the horizontal plane of CBCT, the image of the corresponding tooth position was captured according to the maximum proximal and distal diameter. C) On the coronal plane, the horizontal line passes through the enamel cementum boundary, and the vertical line is the line between the incisal midpoint and the apical point. (D) Captured sagittal plane image of corresponding tooth position.


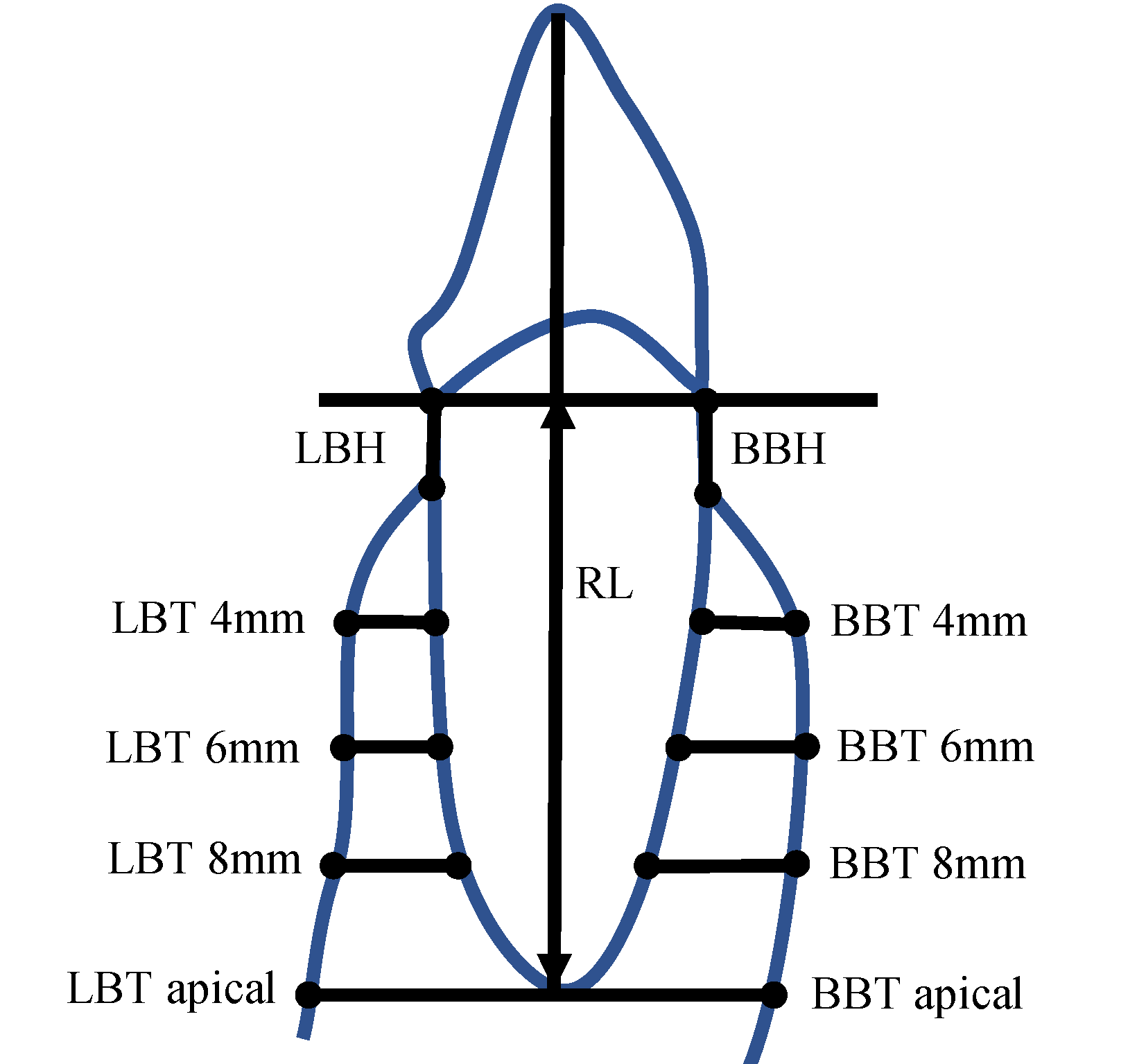


**Supplementary Figure 2**. The bone thickness performed at four distinct distances from the CEJ: 4 mm, 6 mm, 8mm, and apical point. Bone height refers to the distance from alveolar crest to CEJ. RL(root length): the distance from the midpoint of the lip-lingual CEJ line to the apex of the root.


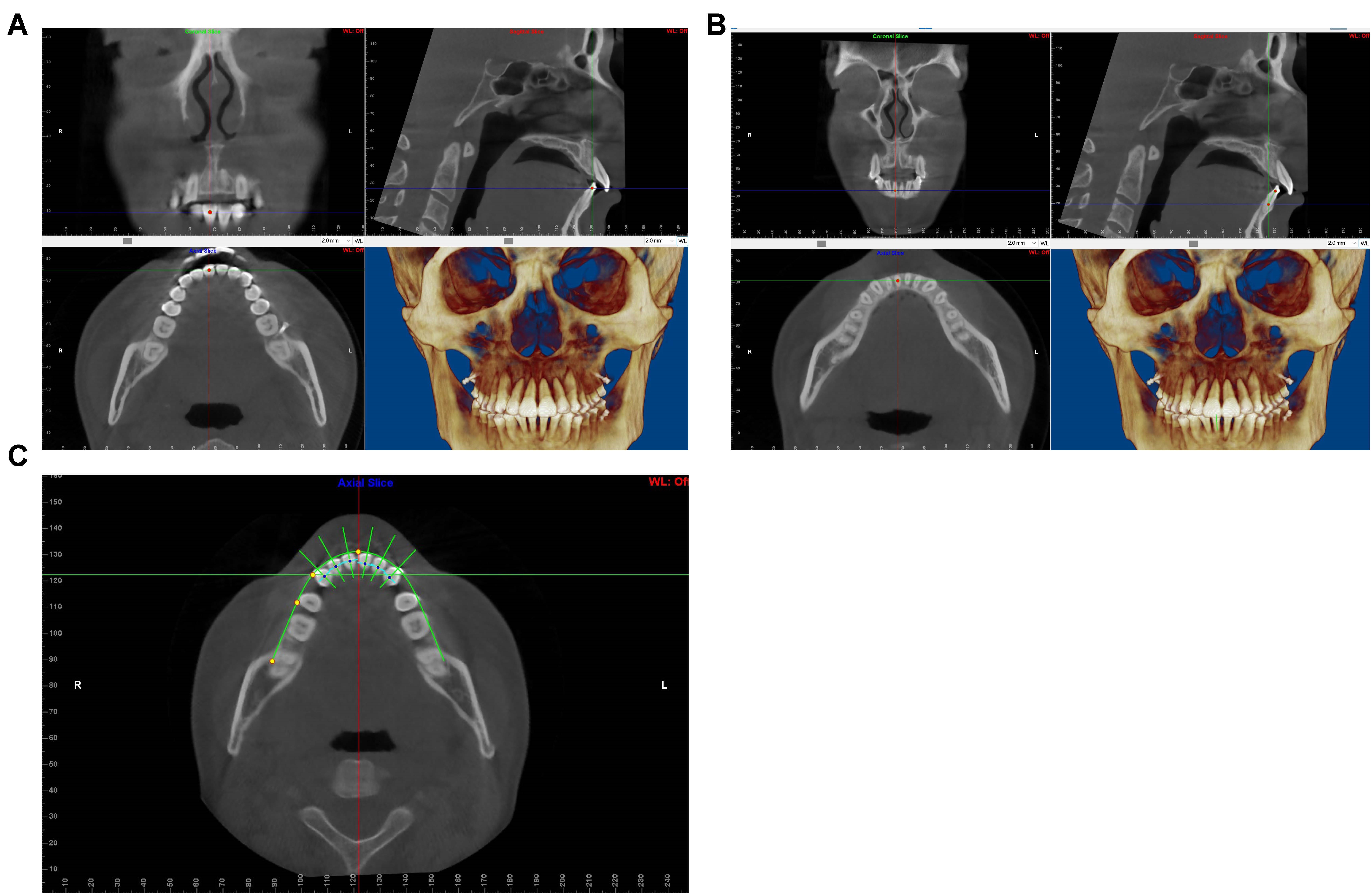


**Supplementary Figure 3**. (A) The positioning of the crown center point (C point). (B) The positioning of the root center point. (C) The mandibular arch was determined on the cross-sectional plane at C point level, with its outline formed by connecting the midpoint between teeth 31 and 41, tooth 43, tooth 45, and the distal point of tooth 47.

**Supplementary Table1.** Basic patient information.

| **Group** | **PAOO group**  **(n=15)** | **Conventional orthodontic group**  **(n=15)** | **Chi-square/*t*** | ***P*** |
| --- | --- | --- | --- | --- |
| **Gender** |  |  |  |  |
| Male (n, %) | 5 (33.3%) | 6 (40.0%) | 0.144 | 0.705 |
| Female (n, %) | 10 (66.7%) | 9 (60.0%) |  |  |
| **Age (years)** | 26.20±6.19 | 27.67±6.39 | -0.639 | 0.528 |
| **Periodontal status** |  |  |  |  |
| Healthy periodontium (n, %) | 11 (73.3%) | 9 (60.0%) | 0.600 | 0.439 |
| Periodontitis under stable control (n, %) | 4 (26.7%) | 6 (40.0%) |  |  |
| **Appliance type** |  |  |  |  |
| Fixed appliances (n, %) | 9 (60.0%) | 6 (40.0%) | 1.200 | 0.273 |
| Invisible appliances (n, %) | 6 (40.0%) | 9 (60.0%) |  |  |
| **Extractions of lower permanent teeth** |  |  |  |  |
| Yes | 10(66.7%) | 8(53.3%) | 0.556 | 0.456 |
| No | 5(33.3%) | 7(46.7%) |  |  |
| **SNA (°)** | 82.45±3.59 | 81.98±3.84 | 0.344 | 0.733 |
| **SNB (°)** | 75.30±4.01 | 75.45±3.54 | -0.111 | 0.912 |
| **ANB (°)** | 7.15±1.14 | 6.59±0.92 | 1.449 | 0.158 |

PAOO: Periodontally accelerated osteogenic orthodontics.

**Supplementary Table 2.** The changes in torque and tip of different tooth site in patients with skeletal Class II malocclusion at 1-year following PAOO treatment.

| **Tooth** | | **T0** | **T1** | **Δ** | ***P*** |
| --- | --- | --- | --- | --- | --- |
| **Tip (°)** | **Central incisors** | -0.61±2.97 | 0.7±1.49 | 1.31±2.04 | **0.001**** |
|  | **Lateral incisors** | -0.05±4.35 | 0.50±2.33 | 0.55±2.37 | 0.232 |
|  | **Canines** | 7.13±4.13 | 5.80±2.66 | -1.33±4.07 | 0.083 |
| **Torque (°)** |  |  |  |  |  |
|  | **Central incisors** | 31.11±3.38 | 28.76±2.42 | -1.37±2.63 | **0.000***** |
|  | **Lateral incisors** | 29.20±2.93 | 27.49±2.73 | -1.71±1.48 | **0.000***** |
|  | **Canines** | 23.49±3.64 | 22.12±2.22 | -2.35±2.42 | **0.008**** |

**:*P* < 0.01, ***: *P* < 0.001. Δ: T1-T0.
